# Sub-lethal concentrations of neonicotinoid insecticides at the field level affect negatively honey yield: Evidence from a 6-year survey of Greek apiaries

**DOI:** 10.1371/journal.pone.0215363

**Published:** 2019-04-25

**Authors:** Robert G. Chambers, Konstantinos Chatzimichael, Vangelis Tzouvelekas

**Affiliations:** 1 Department of Agricultural and Resource Economics, University of Maryland, Symons Hall College Park 2105, MD, United States of America; 2 Faculty of Management and Economics, Cyprus University of Technology, Sp. Araouzou 115, Limassol, Cyprus; 3 Department of Economics, University of Crete, Gallos University Campus, Rethymno, Greece; Bangor University, UNITED KINGDOM

## Abstract

The threats posed by neonicotinoid insecticides to bee populations have been the focus of considerable research. Previous work has shed new light on the effects of neonicotinoids on bees by uncovering pathways through which neonicotinoids affect bee population dynamics and the potential interactions they have with exogenous stressors. Yet, little is known about whether these effects translate in a field-relevant setting to substantial losses in honey yields for commercial beekeepers. Here, we used data from a 6-year survey of 60 apiaries in Greece and economic modelling to assess at the field level the effects of neonicotinoid insecticides on honey production. Based on production function estimates, we found that sub-lethal concentrations of two widely used neonicotinoid insecticides (imidacloprid and thiamethoxam) detected in the nectar of flowers resulted in substantial losses in honey production for commercial beekeepers in our sample. By simulating a scenario with ideal pathogenic and environmental conditions, we found that the magnitude of the neonicotinoid effects decreases significantly under ideal conditions providing evidence for possible synergies at the field between neonicotinoids and environmental and pathogenic factors. Moreover, in a replicated study with grouped apiaries, we found evidence that the marginal effects of neonicotinoids on honey production vary across apiaries facing different conditions.

## Introduction

Apiculture is a vital part of the agricultural economy in many developed and developing countries [1]. According to the FAO, the total number of managed honeybee colonies worldwide was 90.4 million in 2016. Those colonies yielded approximately 1.8 million tonnes of honey production with a gross value of approximately 6.4 billion US dollars [2]. Thus, any threats to apicultural production could have serious consequences for agricultural economy and the livelihoods of thousands of professional and semi-professional beekeepers worldwide [1, 3].

Neonicotinoid insecticides, widely used to manage crop pests, have been widely perceived as a threat to honeybee populations [4–8] and therefore for apicultural production [9]. Although neonicotinoids are not commonly encountered at lethal doses in the field, recent studies have shown that exposure to sub-lethal concentrations distort bee population dynamics by impairing worker bees’ homing ability [10, 11], impairing foraging activity [5, 12], and reducing colonies’ overwinter survival [13, 14] and reproductive success [6, 15]. Neonicotinoids have also been shown to interact with infectious organisms [7, 16, 17], food stress [7], and local conditions [14] to produce negative outcomes for bees.

However, although previous work has significantly advanced our understanding on the effects of neonicotinoids, most of it has focused on the direct effects on bees themselves [18, 19] and not on the indirect effects on honey yields. Equally important, most research was conducted in laboratory or semi-field settings that are not representative of production conditions actually faced by commercial beekeepers. Thus, the degree to which neonicotinoids can decrease commercial honey production, either on their own or synergistically with environmental and pathogenic factors, remains largely unstudied and thus unknown. A quantitative assessment of those effects in a field-relevant setting is needed to enhance our knowledge base and to inform appropriate responses by policymakers and the public.

In this paper, we use data from a 6-year field survey of 60 apiaries in Greece and economic modelling to assess the effects of neonicotinoid insecticides on honey production. Our study aims to examine the degree to which field-level concentrations of neonicotinoid insecticides in the nectar of flowers result in reductions in honey production for commercial beekeepers. Our study aims also to investigate possible interactions of neonicotinoids with environmental and pathogenic conditions in the apiaries and quantify their effects on honey yields.

## Data and Model Description

### Ethics statement

Endangered or protected species were not used in this study.

### Data Description

We investigated the effects of neonicotinoid insecticides on honey production levels using field data for commercial beekeepers. The data involved 60 randomly selected commercial apiaries located in 10 spatially separated (> 24 km) farming-intensive areas on the island of Crete in Greece (6 apiaries per area).

The apiaries and the surrounding landscapes were inspected at the beginning and the end of the honey season (May and October, respectively) for 6 consecutive years from 2006-2011. Permissions were obtained from beekeepers and land owners to conduct the study. In each inspection, samples of flower nectar were taken from multiple spots within a 2 km distance from the apiaries that covers the likely foraging range of honeybees [20]. The sampling spots were selected based on the number of visits of honeybee foragers at flowers accounting thus for possible preferences of foragers for foods containing neonicotinoid residues [21]. At the first inspection of each season (May), on-site measurements on honeybee populations were made on 4-18 randomly selected hives per apiary. Adult bee and brood comb samples were also taken from the selected hives to be tested for the presence of common pathogenic honeybee parasites frequently encountered in Greek beekeeping [22]. At the time of the second inspection (October), information on seasonal honey production volumes and input usage were retrieved directly from beekeepers’ accounting books. In addition, semi-structured interviews were conducted with beekeepers about beekeeping and hive relocation practices used (Details on study design and measurement methods used are presented in the Supporting Information section).

Adult bee and brood comb samples were tested in specialized biology laboratories for the presence of common honey bee infectious agents. Molecular and electron microscopy analysis indicated negative and low-positive samples of *Nosema* apis (Cp = 39.4 ± 0.4), *Nosema* ceranae (Cp = 39.1 ± 0.3), CBPV (Cp = 37.4 ± 0.5), DWV (Cp = 38.8 ± 0.2), ABPV (Cp = 39.9 ± 0.1), and SBV (Cp = 39.8 ± 0.1). On the other hand, 97% of the adult bee samples were diagnosed as positive to Varroa destructor (Varroa mite) with a mean Cp = 17.68 ± 1.6 (Mean Crossing point value ± s.d.). Therefore, only mite infestation was considered in the analysis as the only infectious pathogen traced at significantly high levels.

The samples of nectar were analyzed in a general chemical state laboratory (Laboratory of Analytical Chemistry of the University of Crete) for the presence of 5 neonicotinoid compounds: imidacloprid, thiamethoxam, clothianidin, acetamiprid and thiacloprid; and a pyrethroid: Λ-cyhalotrin. All samples were negative to clothianidin and Λ-cyhalotrin. Hence, four systemic compounds of neonicotinoids (imidacloprid, thiamethoxam, acetamiprid and thiacloprid) were detected in the samples. Acetamiprid and thiacloprid were traced at very low proportions (< 1%) and therefore were not considered in the analysis. Besides being traced at insignificant levels, these two compounds have been shown to result in lower acute toxicity for bees compared to imidacloprid and thiamethoxam [23, 24]. Excluding them should have a minor quantitative influence on the study findings.

**Fig 1:**
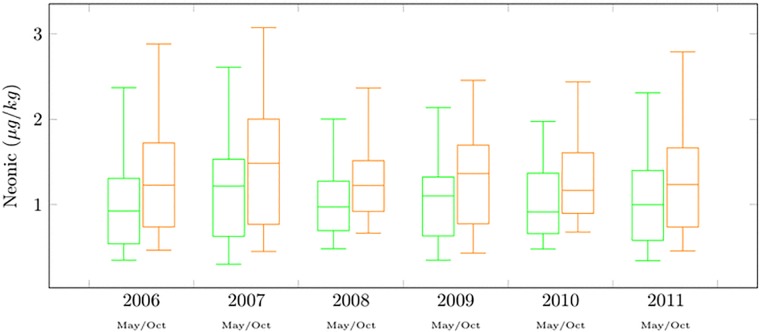
Neonicotinoid concentrationsw. The box-plots provide information about neonicotinoid concentrations levels in nectar from all areas sampled, pooled according to years and seasons. Green and orange lines refer to spring and autumn seasons, respectively.

Imidacloprid and thiamethoxam elicit similar toxicity effects per concentration unit [24] which allows their direct aggregation to construct an additive measure of neonicotinoid concentration. The two compounds were detected together in concentrations between 0.377 *μg*/*kg* and 2.842 *μg*/*kg* with a mean value of 1.386 ± 0.6 *μg*/*kg* (mean ± s.d.). These values are well below the documented lethal-dose levels (LD50 <) but high enough to be suspected for sub-lethal effects [17]. Analyzing the temporal variation and range of the neonicotinoid levels (Fig 1), our data provided evidence for increased accumulation of neonicotinoids in the natural habitat of honeybees between May (1.241 ± 0.5 *μg*/*kg*) and October (1.530 ± 0.6 *μg*/*kg*) implying possible chronic exposure leading to delayed effects over the honey season [25]. Alternatively, this result could be attributed to a more intensive use of neonicotinoid insecticides by farmers later in the season. However, there was no indication that farmers in the surrounding areas had applied insecticides shortly before the collection of nectar samples. Moreover, data analysis provided no indication about the persistence of neonicotinoids in the environment over winter periods. The later result could be attributed to decreases in insecticide use intensity during the winter seasons and to intense rainfalls commonly occurring in winter months which may washed neonicotinoid residues out of honeybees’ habitats.

### Model Description

To assess the effects of neonicotinoids on honey production, we followed a two-step modelling strategy. First, using the concept of a damage function borrowed from the extensive damage and control literature [26–28], we modeled the effects of neonicotinoids on the biological process of honeybees. Second, bee density composed of the initial bee population and the damage function was incorporated into an economic honey-production model. Using the sample data, the model was parametrically estimated in one stage (Details on the proposed model are presented in the Supporting Information section).

Neonicotinoids have been shown to act both in isolation and in synergy with other factors [5, 11–12, 14, 29]. Thus, both neonicotinoids alone and their interactions with mite infestation, food resources, and weather conditions were included in the damage function. However, the own terms of the later set of factors (mite infestation, food resources, and weather conditions) were not included into the specification of the damage function due to important multicollinearity issues. The consequence is that our results may reflect a higher-bound estimate of the interactive effects of neonicotinoids on honeybee population and honey production since the corresponding interaction terms may absorb also part of the direct effects of these factors. In addition, other factors including bee genetics, removing strategy of livestock [1], and beekeeper’s education [30] are known to influence colony losses and therefore should be included into the damage function. However, these factors present zero or little variation across beekeepers in our sample and thus could not be considered in our regression analysis. Other insecticides and pollutants are also known to influence alone or synergetically with neonicotinoids the honeybee populations [31–32]. However, there was no indication that insecticides other than those analyzed in this study were present in the surveyed areas, at least at significant levels (More information about the choice of the compounds analyzed is presented in the Supporting Information section).

## Results and discussion

Our results indicated an average loss of 18.37 ± 8.5% in managed honeybee populations due to neonicotinoid effects (Table 1, upper panel) which is in line with previous findings [3, 7]. That corresponds to annual losses of 1.02 ± 0.6 million honeybees for an average-sized apiary in our sample (average apiary: 133 hives, 5.32 million honeybees). Our results indicated average losses in honey production of 6.78 ± 4.7% which translates into losses of 448.5 ± 31.6 kg of honey per season for an average-sized apiary (Table 1, middle panel). For the whole six year period, honey losses were estimated at 161.5 tonnes for the 60 apiaries analyzed.

**Table 1:** Honeybees and honey production: Damage measures at actual neonicotinoid levels and estimated responses to potential changes in neonicotinoid levels.

	Neonicotinoid Quantiles					Mean Values
	1st	2nd	3rd	4th	5th	
Managed Honeybee Population						
*Estimated Losses*						
Percentage Losses (in%)	9.44	19.54	24.36	18.43	20.06	18.37
Absolute Losses (in 000’s of bees)	636.4	1,142.2	1,351.7	1,159.0	840.8	1,026.0
*Estimated Responses to Changes in Neonics Levels*						
Percentage Response to +1% (in%)	0.100	0.221	0.281	0.208	0.233	0.208
Absolute Response to +0.05 *μg*/*kg* (in 000’s of bees)	75.2	67.6	63.2	44.8	23.1	54.8
Absolute Response to +0.10 *μg*/*kg* (in 000’s of bees)	149.2	134.0	125.5	89.2	46.0	108.8
Honey Production						
*Estimated Losses*						
Percentage Losses (in%)	2.51	6.55	8.64	6.71	9.50	6.78
Absolute Losses (in *kgs* of honey)	218.5	437.4	602.4	458.1	526.0	448.5
*Estimated Responses to Changes in Neonics Levels*						
Percentage Response to +1% (in%)	0.029	0.087	0.118	0.087	0.125	0.089
Absolute Response to +0.05 *μg*/*kg* (in *kgs* of honey)	38.8	35.1	31.0	24.5	19.2	29.7
Absolute Response to +0.10 *μg*/*kg* (in *kgs* of honey)	78.2	70.8	62.6	49.3	38.4	59.9
Area and Apiary Characteristics						
Neonicotinoid Concentration (in *μg*/*kg*)	0.659	1.088	1.325	1.672	2.184	1.386
Apiary Size (in 000’s of bees)	6,291.7	5,570.0	5,396.1	5,702.2	3,650.0	5,322.0
Aridity Index	0.874	0.537	0.518	1.094	0.947	0.794
Relative Humidity (in%)	0.440	0.491	0.508	0.409	0.354	0.441
Winter Precipitation (in *mm*)	381.3	401.9	368.1	429.4	529.6	422.1
Mite Infestation (in 000’s of Mites)	5.73	5.62	5.00	5.50	5.68	5.51

The 60 apiaries in the samples were sorted with an increasing order based on the neonicotinoid concentrations observed in the surrounding areas. Next, they were grouped into five equal neonicotinoid quantiles with the first quantile including the 12 apiaries exposed to the lowest neonicotinoid levels, the second quantile including the 12 apiaries exposed to higher neonicotinoid levels and so on. Annual average values are shown per neonicotinoid quantile.

To determine the responsiveness of honey production to incremental changes in neonicotinoid concentrations, we performed a marginal analysis based on the parameter estimates of the model. We found that, other things equal, a 1 per cent increase in the neonicotinoid concentrations results in losses of 0.208 ± 0.11% and 0.089 ± 0.05% in honeybee population and honey production, respectively. We repeated the marginal analysis in absolute terms assuming incremental increases of 0.05 and 0.10 *μg*/*kg* in neonicotinoid levels. We found the corresponding losses in honey production to be 29.7 ± 15 kg and 59.9 ± 31 kg per season for an average-sized apiary (Table 1, middle panel).

The effects of neonicotinoids on honey production are expected to increase at higher concentrations. But precisely how these effects vary with concentration levels cannot be determined *ex ante*. Therefore, we used our estimated model to identify empirically how honey production responds to increasing the concentration of neonicotinoids. Sample apiaries were sorted by exposure levels detected in the surrounding areas and then grouped into equal neonicotinoid quantiles. The first quantile included the 12 apiaries exposed to the lowest neonicotinoid concentrations, the second quantile included the 12 apiaries exposed to higher concentration levels, and so on.

We found that losses in honey production are correlated to losses in honeybee population in the same quantiles but not with apiary size. We also found that apiaries in the first quantile, which were exposed on average to 0.659 *μg*/*kg* of imidacloprid and thiamethoxam, experienced significantly lower losses in honeybee population and honey production when compared with apiaries in higher quantiles (Table 1). We did not, however, observe significant increasing losses across the remaining four higher neonicotinoid quantiles. These insignificant linear trends might be attributed to differences in environmental and pathogenic conditions across apiaries which may have altered the magnitude of the neonicotinoid effects on honeybee population and honey production. It should be mentioned though that the trends are generally consistent and vary according to residue levels, which is indicative of a cause-effect relationship.

**Fig 2:**
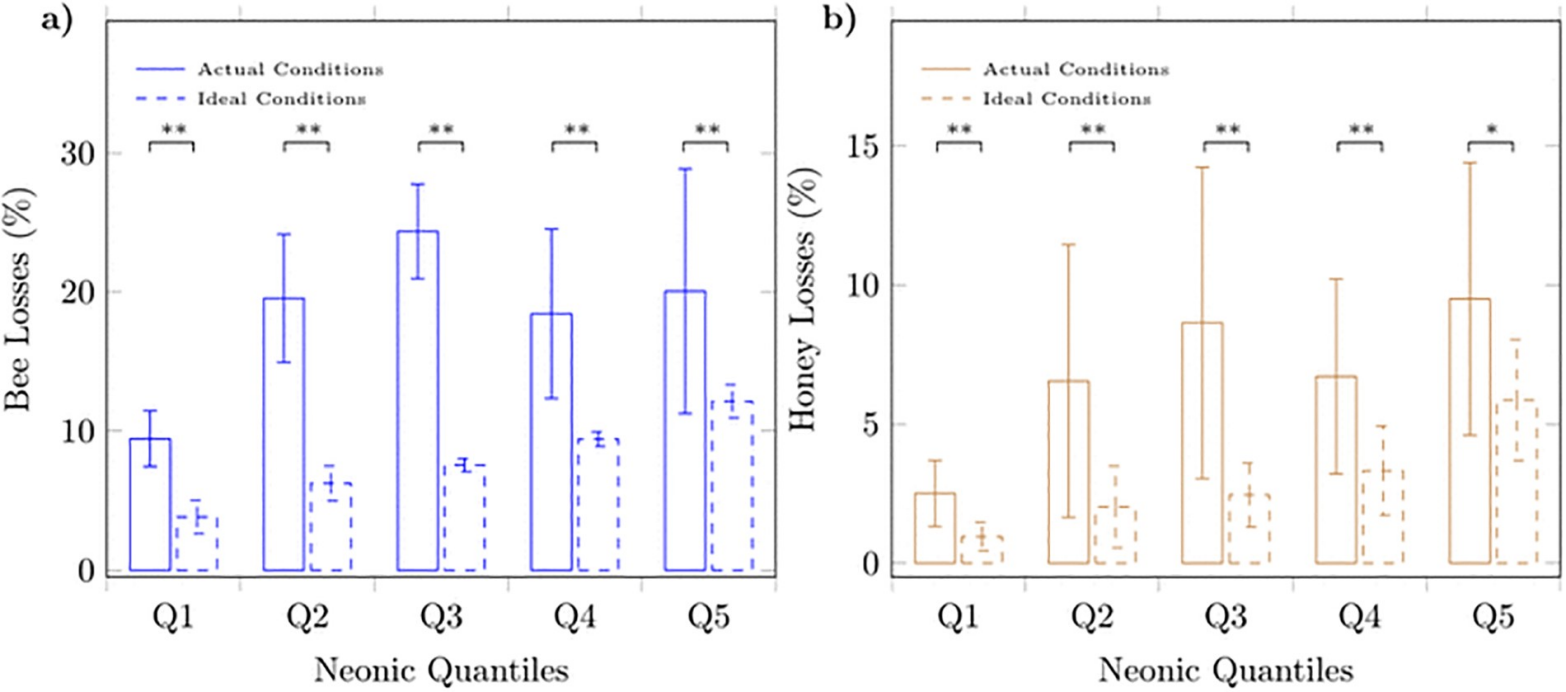
Honeybee and honey losses per neonicotinoid quantile under actual and under ideal conditions. Mean losses in managed honeybee population (**a**) and honey production (**b**) under actual and ideal field conditions. Details about the construction of neonicotinoid quantiles are provided in the caption of Table 1. Solid and stippled lines refer to actual and ideal conditions, respectively. Means ± s.d. are shown separately for every neonicotinoid quantile. Results from one-tailed paired t-test are shown; **p < 0.01, *p < 0.05.

To examine whether our results were sensitive to differences in environmental and pathogenic conditions in the field (Table 1, lower panel), we simulated a scenario in which all sample apiaries are facing equal field conditions. We did so by assigning a predetermined set of fixed values to the condition-related variables of the model. The set of values was determined so as to reflect near-ideal conditions in the apiaries (Ideal conditions: winter precipitation = 520*mm* of rain as a proxy of food resources, aridity index = 0.83 and relative humidity = 58% as proxies of weather conditions, number of mites = 0). Then, we used the estimated model to project responses of honey production to increases in neonicotinoid levels. We found that under ideal conditions, honey losses increase robustly across all neonicotinoid quantiles (Fig 2). We also found honey losses to be considerably smaller compared to those under actual conditions in all five neonicotinoid quantiles (one-tailed paired *t*-test: *t* > 2.17, *df* = 11, *p* < 0.026) providing evidence that the magnitude of neonicotinoid effects may depend upon environmental and pathogenic conditions. This finding suggests the presence of possible synergies at the field between neonicotinoids and environmental and pathogenic conditions.

**Fig 3:**
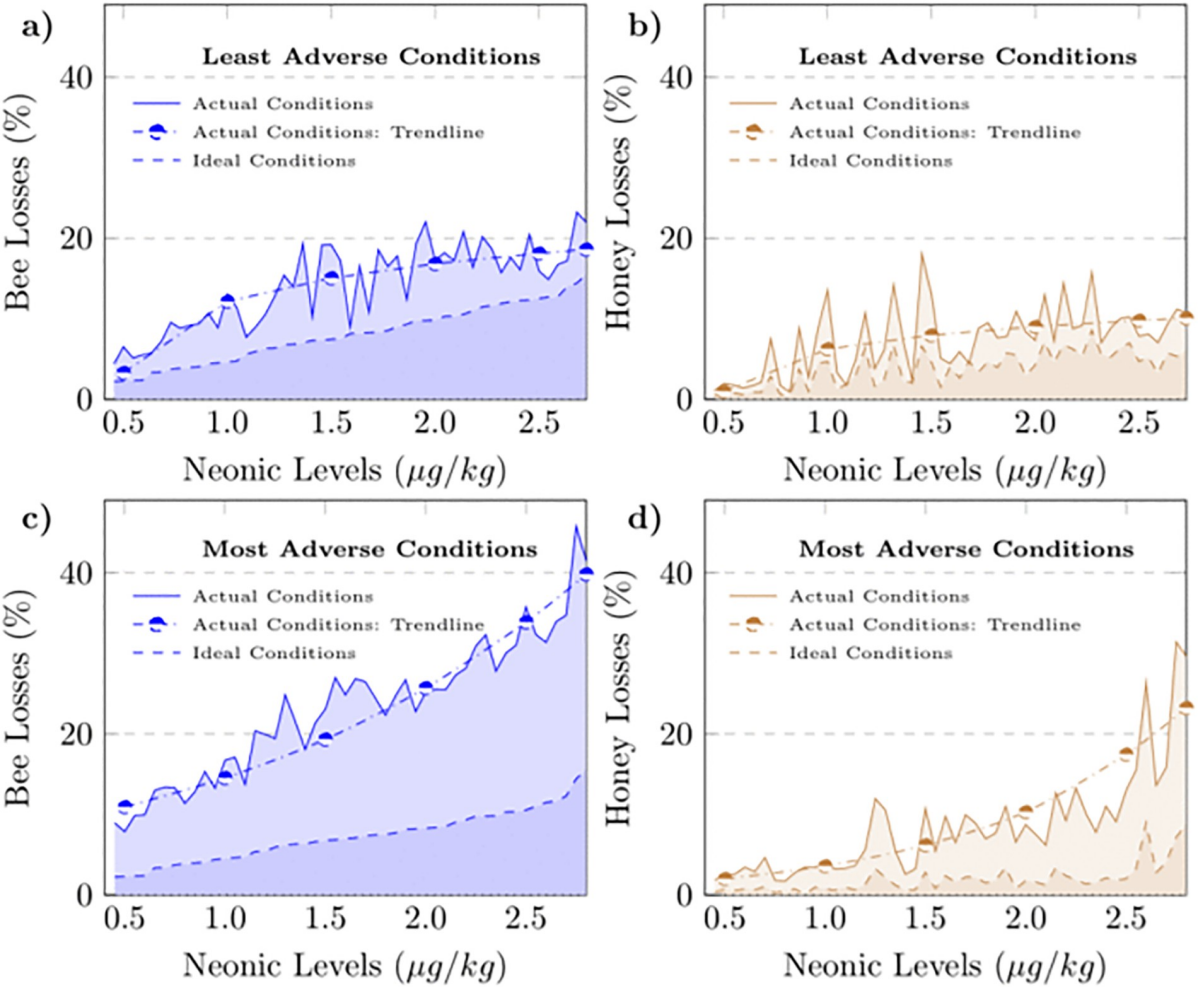
Honeybee and honey losses across neonicotinoid levels for apiaries facing the least and most adverse conditions. Losses in managed honeybee population under actual and ideal conditions for apiaries facing the least adverse conditions (**a**), losses in honey production under actual and ideal conditions for apiaries facing the least adverse conditions (**b**), losses in managed honeybee population under actual and ideal conditions for apiaries facing the most adverse conditions (**c**), losses in honey production under actual and ideal conditions for apiaries facing the most adverse conditions (**d**). Based on the parameter estimates of the damage function and actual data on weather conditions and mite infestation, an index of the overall conditions prevailing at the apiaries every season was constructed. Based on the index, apiaries were classified into two equal groups with the first and second group including the apiaries facing the least and most adverse conditions, respectively. The choice of functional form for the trend lines was based on goodness-of-fit measures. Three alternative functional forms were considered for the approximation of the trend lines, namely, the linear, logarithmic and exponential functional form.

To investigate the extent to which adverse conditions may have increased the magnitude of the neonicotinoid effects, we classified apiaries into two equal groups based on environmental and pathogenic conditions and then replicated the simulation analysis for each group. The first group included the apiaries facing the least adverse conditions and the second group included those facing the most adverse conditions. Under ideal conditions, we found quite similar neonicotinoid effects across the two groups. Under actual conditions, we found significantly higher effects for the second group facing the most adverse conditions (Fig 3). In both groups, neonicotinoid effects were found to increase in general with increasing concentrations. However, the severity of these effects across concentration levels was different between the two groups. Honey losses followed a logarithmic trend with concentration levels in the first group and an exponential trend in the second group implying decreasing and increasing marginal effects, respectively.

To obtain a quantitative assessment of the interactive effects of neonicontoinoids, we conducted a variance analysis within each group considering the mean difference between the honey losses under actual conditions and the honey losses that would have occurred under ideal conditions. Our results indicated that deviations from ideal conditions increased honey losses by 2.53% ± 2.03 for apiaries facing the least adverse conditions and by 5.28% ± 4.60 for apiaries facing the most adverse conditions. Focusing on concentrations higher than 1.5 *μg*/*kg*, we found that the increase in honey losses due to interactive effects were 2.76% ± 2.16 for apiaries facing the least adverse conditions and 8.63% ± 6.40 for apiaries facing the most adverse conditions.

## Conclusion

In this paper, we used data from a 6-year survey of 60 apiaries in Greece and economic modelling to assess the effects of neonicotinoid insecticides on honey production. Our results indicated that sub-lethal concentrations of imidacloprid and thiamethoxam detected in the nectar of flowers resulted in substantial losses in honey production levels for beekeepers in our sample. This finding is important because it improves our understanding of the economic welfare losses associated with neonicotinoid exposure. Our results provided also evidence for possible synergies at the field between neonicotinoids and environmental and pathogenic conditions prevailing at the apiaries. These synergetic effects were found to account for significant losses in the honey yields of beekeepers. However, these results reflect only a higher bound estimate of the interactive effects of neonicotinoids. Finally, our results indicated decreasing marginal effects of neonicotinoids on honey production for beekeepers in our sample facing the least adverse conditions and increasing marginal effects for beekeepers facing the most adverse conditions. This result indicates that potential increases in neonicotinoid levels are likely to lead to higher losses in honey production under adverse conditions, especially if neonicotinoids are present at high concentrations.

## Supporting information

S1 File Supplementary information. (PDF)

S1 Table Summary statistics of the variables. (PDF)

S2 Table Parameter estimates of the translog production function. (PDF)
