# Supplementary material for: Sub-lethal concentrations of neonicotinoid insecticides at the field level affect negatively honey yield: Evidence from a 6-year survey of Greek apiaries
Source: PLoS One. 2019 Apr 25;14(4):e0215363. doi: 10.1371/journal.pone.0215363 (PMC6483167; doi:10.1371/journal.pone.0215363)
Supplement: S1 File — (PDF) [file pone.0215363.s001.pdf]

Supplementary Information

Model setup

A two-step approach was adopted for modelling the effects of neonicotinoids on honey production. In the first step, neonicotinoids, neonicotinoids $\times$ mite infestation, neonicotinoids $\times$ winter precipitation, neonicotinoids $\times$ humidity, and neonicotinoids $\times$ aridity were embedded into a damage function defined in generic form as [1–3] $\phi_{it}\left(z_{it}, \boldsymbol{s_{it}}; \boldsymbol{\alpha}\right)$, where i indexes the apiaries, t indicates the time period, $\phi : \Re_{+}^{5} \rightarrow [0, 1]$ is the damage function having the properties of a cumulative probability distribution, $z \in \Re$ denotes neonicotinoid concentration, $s \in \Re_{+}^{4}$ is the vector of exogenous variables including mite infestation levels proxied by the number of mites per hive, food resource availability proxied by winter precipitation, and weather conditions proxied by relative humidity and the aridity index and α's are parameters to be estimated. Bee density, $\tilde{b}$, was defined in each apiary as:

$$\tilde{b}_{it} = b_{it}\left[1 - \phi_{it}\right] \tag{1}$$

where b_{it} is bee population at the beginning of the honey season. In the second step, bee density was embodied within a honey production function defined as: $y_{it} = f\left(\tilde{b}_{it}, \boldsymbol{x_{it}}, t; \boldsymbol{\beta}\right)$, where $y \in \Re$ is output, $f : \Re_{+}^{j+2} \rightarrow \Re_{+}$, is a continuous and, strictly increasing, twice differentiable concave production function, representing maximal output from honeybee density and productive inputs given the exogenous variables and the available technology, $x \in \Re_{+}^{4}$ is a vector of productive inputs including veterinary expenses, intermediate inputs, family labor, and capital stock, and β's are parameters to be estimated. Summary statistics of the variables are presented in S1 Table.

Functional forms

The following exponential functional specification embodying the biological relationships involved in the growth and development of honeybee populations was used to approximate the damage function [2]:

$$\phi_{it} = 1 - exp\left(-\alpha_z z_{it} - \sum_q a_{zq} z_{it} s_{qit}\right) \tag{2}$$

For the approximation of the production function, we used the following flexible transcendental logarithmic (translog) functional specification [4]:

$$\begin{aligned} \ln y_{it} = {} & \beta_0 + \beta_b \ln \tilde{b}_{it} + \sum_j \beta_j \ln x_{jit} \\ & + t\left[\beta_t + \frac{1}{2}\beta_{tt} t + \beta_{bt} \ln \tilde{b}_{it} + \sum_j \beta_{jt} \ln x_{jit}\right] \\ & + \frac{1}{2}\left[\beta_{bb} \ln^2 \tilde{b}_{it} + \sum_j \sum_\rho \beta_{j\rho} \ln x_{jit} \ln x_{\rho it}\right. \\ & \left. + \sum_j \beta_{bj} \ln \tilde{b}_{it} \ln x_{jit}\right] + v_{it} \end{aligned} \tag{3}$$

where $v_{it} \sim N\left(0, \sigma_v^2\right)$ is a normally distributed error term capturing omitted explanatory variables and measurement errors in the variables. Upon substituting (2) into (1) and then into (3), the resulting model was estimated in one stage providing estimates for α and β parameters. Parameter estimates of the model are reported in S2 Table.

Measurement of neonicotinoid effects

Measurements on the percentage losses in honeybee populations were obtained directly by the fitted values of the damage function ($\hat{\phi}_{it}$). The number of bees lost (absolute losses) was computed as $b_{it} \times \hat{\phi}_{it}$. Honey losses in each apiary were measured as the maximal possible honey production that would have been realized in the absence of neonicotinoids minus the maximal possible honey production in the presence of neonicotinoids at their actual levels. The later was obtained by the fitted values of the production function and the former by the fitted values of the production function after imposing z=0 in relation (2). To project losses in bee population and honey production under ideal conditions, we assigned a set of fixed values to the condition-related variables included in vector $s \in \Re^4_+$ and then repeated the measurements as described above. The set of fixed values was determined so as to reflect near-ideal conditions in the apiaries.

Survey design

The survey included 60 randomly selected apiaries owned by professional beekeepers located in ten spatially separated areas (>24km) in the Western part of the island of Crete in Greece. An equal number of apiaries was selected from each area resulting in 6 apiaries per area. The 10 areas were selected randomly from a total of 38 areas in the western part of the island where professional beekeepers are known to maintain their apiaries. A pilot survey was conducted in August 2005. In the course of the pilot survey, the areas surrounding the apiaries were inspected and information on the spatial characteristics, geographical proximity and floral diversity of the areas were recorded. Areas' inspection revealed areas that were very homogeneous over these characteristics and closely located apiaries typically adjacent to each other. During the pilot survey, all apiary owners were interviewed and agreed to participate in the survey. Preliminary interview results indicated that beekeepers were using similar relocation practices including three moves during the year in the middle of October (to overwinter), beginning of March (to restore colonies' strength) and beginning of May (for the honey harvesting period). Hence, beekeepers were highly homogenous with respect to the relocation practices used indicating that this variable is constant in our sample. Preliminary interview results indicated also that the first exposure of honeybees to neonicotinoids during the year was in the beginning of the honey season when they were relocated to the apiary sites. Before this move, all beekeepers indicated that they maintained the hives in non farming areas (from October to April). In addition, interview results indicated that beekeepers commonly perform hive splitting tasks shortly before the relocation of hives to the apiary sites for the honey season. All apiary owners agreed to inform in advance the survey team about the hive relocation dates. Finally, in the course of the pilot survey, 10-15 crop farm operators from each area within a distance of 5km from the apiaries were interviewed about the types of insecticides used. Based on this information, the compounds of neonicotinoids which were likely to be present in the surrounding areas were identified. The main survey commenced in 2006 and took place for 6 consecutive years until 2011 that is shortly before EU imposed a moratorium in the use of neonicotinoid insecticides [5]. In the course of the survey, all 60 apiaries in the sample were inspected twice per year at the beginning (28 Apr - 15 May) and the end (28 Sep - 15 Oct) of the honey season, respectively. In the course of the first inspection of each season, area-specific measurements on neonicotinoid concentrations were performed. Moreover, at each apiary, measurements on honeybee populations were performed and brood comb samples were collected from hives. In the course of the second inspection of each season, area-specific measurements on neonicotinoid concentrations were repeated. Moreover, beekeepers' accounting books were reviewed and personal interviews were performed with apiary owners. In addition, four visits were made to all

apiaries in the middle of the seasons at the beginning of months June, July, August and September and measurements were performed on mite infestation levels. The survey was partly supported by the Specific Targeted Research Sixth Framework EU Project TEAMPEST under contract number 212120 and was conducted in cooperation with National Agricultural Research Foundation (NAGREF).

Nectar samples

Nectar samples from flowers and herbaceous plants were collected twice per year between 1 May and 15 May and between 28 Sep and 12 Oct. In each area, 12 nectar samples were taken in the course of each inspection from different spots within a $2km$ distance from the apiaries. This distance corresponds to two times the average honeybee foraging range, $1km$ [*6*]. Hence, contaminated resources located farther away from the average honeybee foraging range have been also taken into account. The sampling spots were selected based on the number of visits of honeybee foragers at flowers. Specifically, nectar foraging in each area was observed for two hours per day within a period of 5 days. Observation periods were from 9:00-10:00 and from 16:30-17:30. Observations were made by 12 observers, each assigned to monitor fields of about 1 km^2. During the first day, the landscape within each field was inspected by the corresponding observer and all floral grasslands were marked with cable ties. In the following two days, the marked grasslands were observed and the most visited grassland within each field of responsibility was identified for subsequent observation. The most visited grassland was divided into sub-fields of $40m^2$. During the following two days, the sub-field exhibiting the highest visitability was identified and tagged for further observation. All flowers within the sub-field were marked with numbers. In the course of the fourth and fifth day, the number of honeybee visits at each flower was recorded within the tagged sub-field. Observers considered as visits only those lasted more than 5 seconds. The average time spent by honeybees per visit was measured at 8.1$\pm$ 1.4 seconds with very little variation across areas. Nectar samples were next collected from the most visited plant in each sub-field resulting in 12 samples from each area. No process was used to validate that honeybees observed were from the surveyed apiaries. However, this is not expected to introduce important bias in the measurements since the visits were used as an instrument to select the sampling spots. Alternatively, sampling spots could have been randomly selected. At least, 1.5 grams of nectar were collected per sample in the course of each inspection indicating a minimum of 18 grams of nectar from every area. To examine if the selection of different spots would result in different measurements in neonicotinoid concentrations, we performed a set of post hoc distributional tests on concentrations detected in area-specific multiple spot samples. Statistical testing results using the Kolmogorov-Smirnov test failed to reject the hypothesis of a uniform distribution of neonicotinoids across each inspected area ($D < 0.25$, $n = 12$, $\alpha = 0.05$) suggesting possibly equally contaminated fields. This result indicates that selecting different sampling spots within each area would not be likely to make any statistically significant difference in the measurement of neonicotinoid concentrations.

Neonicotinoid concentrations

Nectar samples were analyzed in the Laboratory of Analytical Chemistry of the University of Crete (Division of Environment and Analytical Chemistry, Department of Chemistry, University of Crete, Heraklion City, Island of Crete, Greece). Nectar samples were analyzed for the presence of 5 neonicotinoid compounds: imidacloprid, thiamethoxam, clothianidin, acetamiprid and thiacloprid; and a pyrethroid: Λ-cyhalotrin. Concentrations were quantified using liquid chromatography with tandem mass spectrometry (*7*). Neonicotinoid levels detected in the 12 samples from each area were averaged to

define the mean concentration of the area (Limits of detection: 0.1-10$\mu g/kg$). Since the measurements were referring to two points of time within each season (beginning and end of the honey season), the two means were also averaged and the resulting figure was used to determine neonicotinoid levels within each area and for each season. The distance of the sampling spot from the apiaries was not considered when calculating the mean concentration of neonicotinoids in each area. This is because concentrations detected in each area were found to follow a uniform distribution. Therefore, down-weighting concentration levels by distance would not make any difference in the measurements.

Adult bee and brood comb samples

Adult bee and brood comb samples were collected from inside the hives within a period of 3 days from 08 May to 15 May. Between 4 and 18 samples were collected from different hives within each apiary depending on the size of the apiary. This corresponds to the 5-10% of the total number of hives in each apiary. The selection of hives was blinded and was repeated in each season. Therefore, different hives were likely to be considered every season. In addition, adult bee and brood comb samples were collected in the middle of the season within a period of 2 days between 15 July and 30 July to identify possible changes in pathogenic conditions in the apiaries. This sampling process was of a smaller scale involving 1 to 4 hives in each apiary. All samples were tested in specialized biology laboratories for the presence of Nosema apis, Nosema ceranae, Chronic bee paralysis virus (CBPV), Acute paralysis virus (ABPV), Deformed wing virus (DWV), and Sacbrood virus (SBV) using one-step real time RT-PCR for viruses detection and RFLP-PCR for Nosema speciation [*8*]. Scanning electron microscopy was also performed on samples for detection of honeybee mites. LightCycler software was used to analyze acquired fluorescence data and the crossing point (Cp), was determined automatically based on the Fit Points method. Samples exhibiting a crossing point (Cp) lower then 35 were defined as positive. Samples exhibiting a Cp between 35 and 40 were defined as low positive while those exhibiting a Cp equal to 40 were defined as negative. The Cp value is the cycle at which fluorescence achieves a defined threshold and corresponds to the cycle at which a statistically significant increase in fluorescence is first detected. Specifically, a threshold line was defined above the noninformative fluorescent data. Next, data points from the log-linear region of the fluorescent curves were used to generate the "best-fit" regression line, namely, crossing line. The intersection of the fluorescent curve with the crossing line was used to determine the fractional cycle number of the crossing point.

Honeybee population

Bee population was measured by visual estimation of adult workers density on comb sides [*9,10*]. At each apiary, 4 to18 hives were blindly selected for observation. The exact number of hives was determined by the size of the apiary ensuring that at least the 5% of the hives in each apiary were observed. Selected hives were opened and the combs were sequentially removed. Next, observers visually estimated the percentage of the comb surface covered by adult workers using a pre-marked grid. All visual observations were initiated at 06:30 and completed at 07:15. In cases that the time window was not sufficient to complete all observations in an apiary, the task was continued the following day. All observations were made between 01 May and 19 May. The exact date of observation depended on the relocation date of the hives to the area. Specifically, all observations were made at least one day and at most three days after hives relocated to the apiary sites for the honey season to allow honeybees sufficient time to recover from moving stress [*11*] and minimize exposure time to neonicotinoids since both could potentially affect the measurements. The observed density on comb sides was used to extrapolate the number of bees in each hive [*9*]. The estimated populations in each hive

were averaged to determine a point estimate of the mean population in each hive. This figure was multiplied by the number of hives in the apiary to proxy the total number of honeybees per apiary. Confidence intervals were build using t-distribution statistical values.

Honey production

Information regarding honey production levels and input usage was retrieved directly from beekeepers accounting books. Accounting books were reviewed in the presence of apiary owners within a period of 2 days between 01 Oct and 12 Oct. Honey production level was determined as the total volume of honey harvested within the season and was measured in kgs. The quantity of honey left in the hives for the needs of honeybees after each harvest was not considered in our analysis due to practical reasons associated with measurement difficulties. The quantity of honey left in the hives was typically predetermined and practices used with respect to this procedure were quite similar across all beekeepers, therefore this exclusion was not expected to have any significant effect on the results. The productive inputs considered in the analysis were intermediate inputs, veterinary expenses, labor input, and capital stock. Intermediate inputs consisted of goods and materials used during the season. These included fuel, electric power, storage expenses, and feeding expenses. The different categories were aggregated into a single input index using the *Tornqvist* approximation to the Divisia index. In particular, national price indices for fuel, electric power, storage and honeybee feed were used to construct an aggregate price for intermediate inputs using the Tornqvist price index [*12*]. The cost shares of each type of expenses to total expenses were used as weights in the construction of the aggregate price index. Next, the total cost associated with intermediate inputs was divided by the aggregate price index. The resulting figure was used to measure intermediate inputs. Veterinary expenses, also measured in Euros, consisted of expenses on antibiotics and other medication including miticides and expenses on veterinary physicians. Again, the *Tornqvist* approximation was used to aggregate the above categories. Family labor, measured in working hours, included total family hours (bee farm owner and family members) devoted to working tasks associated with beekeeping. Capital stock measured in Euros included the value of hive boxes and hive frames, smokers and other hive tools, clothing equipment and storing cans. The computation of the capital stock was based on the perpetual inventory method assuming a depreciation rate of 8%.

Mite infestation

Mite infestation at each apiary was proxied by the total number of varroa mites per hive. Four measurements on mite infestation took place during each season between 1-5 June, 1-5 July, 1-7 August, and 1-5 September. At each apiary, 4 to18 hives were blindly selected to be used for measuring mite infestation. The number of mites was estimated using the ”sticky board” test method [*13*]. Specifically, a sticky board was placed on the bottom of the hive for 48 hours. The number of dead mites falling to the bottom of the hive was next counted. Based on the number of mites found on the sticky board and the mortality rate of mites, their total number was extrapolated. In cases that acaricides had been used by beekeepers to deal with mites, the efficiency rate of the miticide was also accounted for by extrapolating the total number of mites in hive [*13, 14*]. The estimated number of mites in each hive were averaged to determine a point estimate of the mean number of mites per hive. Since the measurements were referring to four different points of time within each season, the four means were also averaged and the resulting figure was used to determine mite infestation levels within each apiary and for each season. Confidence intervals were built using t-distribution statistical values.

Food resources

Because areas analyzed were homogenous in terms of altitude, soil conditions, and flora diversity, food resource availability was proxied solely by winter precipitation as the most important factor accounting for differences in flowering time and nectar richness of wildflowers and herbs [*15*]. The index was constructed over the period from October to April and it was measured in millimeters of rain. Measurements of the winter precipitation were obtained from the meteorological stations located throughout the island producing continuous spatial grids of weekly air temperature and precipitation. Up to a certain threshold, increases in winter precipitation levels contribute positively to soil fertility [*16*] and flowering time [*15, 17*] of plants leading to rich floral resources for honeybees during the honey season. However, because extreme winter precipitation might have the opposite effect, we initially fitted a quadratic term into the model with respect to winter precipitation variable to test for possible non-linear effects. The associated second order parameter was found statistically insignificant implying that winter precipitation was not exhibiting a certain threshold. Thus, the quadratic term was not considered in the final model.

Weather conditions

Weather conditions in each area were proxied by relative humidity and aridity levels since both weather variables can interact significantly with neonicotinoids influencing the foraging activity of bees. The aridity index was constructed as the ratio of the average ambient temperature over the total precipitation in the area where apiaries were located [*18*]. Both relative humidity and aridity index were computed over the period from 1 May to 12 October. The meteorological data for the weather variables were obtained by the local Meteorological Stations located throughout the island. High rates of relative humidity make heavier the wings of honeybees which in turn implies that honeybees need to consume more energy for their flights. As a result, the frequency and duration of the flights are decreased when relative humidity exceeds a certain threshold. In addition, high rates of relative humidity act negatively in the concentration of sugars in the nectar of flowers which in turn reduces the attractiveness of food resources for bees. With food resources being less attractive, honeybees reduce their flights [*19, 20*]. In overall, high rates of relative humidity rates above a certain threshold are expected to affect negatively the flight activity of honeybees. On the contrary, low rates of relative humidity have no direct effects on the flight activity of Apis species but can increase the attractiveness of resources. Ambient temperature and summer precipitation are both related with the duration and frequency of foragers' flights. Up to a certain threshold, increases in ambient temperature decrease the time and energy required by honeybees to elevate their thoracic temperature before flight contributing thus positively to the foraging activity of bees [*21*]. Similarly, low summer precipitation levels increase the frequency of foragers' flights. Therefore, increases in aridity levels up to a certain threshold are expected to enhance foraging. However, extreme temperatures and very low summer precipitation levels might have the opposite effect on flight duration by increasing rapidly the body heat of bees during flight and reducing the attractiveness of flowers. Hence, increases in aridity levels above a certain threshold are expected to contribute negatively to flight duration. To test for such non-linear effects, we added two quadratic terms into our model with respect to relative humidity and aridity variables. However, the associated second order parameters were found statistically insignificant implying that weather conditions were not exhibiting a certain threshold. Thus, the quadratic terms were not considered in the final model.

Ideal Conditions

Ideal conditions were determined within the topographic and vegetation characteristics of the areas where apiaries are located. The study areas are characterized by a semi-arid ecosystem with mediterranean climate, sandy soils, and rich grass- and shrub-lands. Within this mediterranean-type ecosystem, winter precipitation levels of 450mm to 650mm of rain have been shown to optimize the cation exchange capacity of soil and the phenology of flowers leading to high levels of soil fertility and nectar-rich wildflower grasslands during the honey season [*15, 16, 22*]. Hence, winter precipitation was ideally set within this interval to 520 millimeters of rain [*16*]. Flight activity of honeybees has been shown to reach its peak at ambient temperatures between 21 and 26 degrees centigrade with low precipitation levels in the form of light drizzly rains [*23*]. Therefore, ambient temperature and summer precipitation were ideally set within these intervals to 23.3 degrees centigrade [*19*], and 28mm of rain resulting in an aridity index of 0.83. At this temperature, relative humidity was ideally set to 58% [*19*]. The ideal levels for the aridity index above refer to Apis Cerana and not to Apis Mellifera honeybee. The two species are known to have slightly different ecological requirements. Hence, these values constitute an approximation of the ideal conditions rather than an accurate measurement. Mite infestation levels were ideally set to zero.

Statistics

Regression analysis and statistical tests were performed using STATA v14. All variables were normalized by their mean value before regression analysis to avoid problems related with measurement units. The model was estimated in one stage with the use of an ordinary least square (OLS) regression procedure. Two alternative functional specifications (Cobb-Douglas and transcendental logarithmic) were initially considered for the approximation of the production function. The former is a special class of the latter that can be arrived at by imposing zero-order conditions on its parameters ($\beta_{bt} = \beta_{jt} = \beta_{bb} = \beta_{j\rho} = \beta_{bj}$, $\forall j, \rho$). The Cobb-Douglas function was statistically tested against the transcendental logarithmic functional form using the log likelihood ratio test (LR test). Based on the testing results ($\chi^2 = 91.43$, $df = 20$, $p = 0.000$), the null hypothesis was rejected at the 1% significance level. Therefore, the *translog* functional specification was used to proxy the honey production technology. The LR test was also employed to statistically test three hypotheses with respect to the features of the honey production technology, namely, the hypothesis of constant returns to scale against variable returns of scale ($\chi^2 = 14.47$, $df = 3$, $p = 0.002$), no technical change against technical change ($\chi^2 = 7.98$, $df = 7$, $p = 0.334$) and *Hicks*-neutral technical change against factor-biased technical change ($\chi^2 = 2.35$, $df = 5$, $p = 0.799$). To consider possible effects of miticides, antibiotics, and feeding expenses on honeybee populations, all three productive inputs were additionally entered alone and interactively with neonicotinoids into the damage function. However, our estimation results did not generate significant coefficients for any of those terms. Hence, the productive inputs were not included in the specification of the damage function.

References

1. R. G. Chambers, E. Lichtenberg, Simple econometrics of insecticide productivity. *Am. J. Agric. Econ.* **76**, 407-417 (1994).

2. G. Fox, A. Weersink, Damage control and increasing returns. *Am. J. Agric. Econ.* **77**, 33-39 (1995).

3. E. Lichtenberg, D. Zilberman, The econometrics of damage control: Why specification matters? *Am. J. Agric. Econ.* **68**, 261-273 (1986).

4. L. R. Christiansen, D. W. Jorgensen, L. J. Lau, Transcendental logarithmic production frontier. *R. Econ. & Stat.* **55**, 28-45 (1973).

5. EU, Regulation (EU) No 485/2013. *Off. J. E.U.* **139**, 12-26 (2013).

6. M. Henry *et al.*, Reconciling laboratory and field assessments of neonicotinoid toxicity to honeybees. *Proc Royal Soc B*, **282**: 20152110 (2015).

7. P. Payá *et al.*, Analysis of insecticide residues using the Quick Easy Cheap Effective Rugged and Safe (QuEChERS) insecticide multiresidue method in combination with gas and liquid chromatography and tandem mass spectrometric detection. *Anal. & Bioan. Chem.* **389**, 1697-1714 (2007).

8. N. Bacandritsos *et al.*, Sudden deaths and colony population decline in Greek honey bee colonies. *J. Inver. Patho.* **105**, 335-340 (2010).

9. M. Burgett, I. Burikam, Number of adult honey bees (Hymenoptera: Apidae) occupying a comb: a standard for estimating colony populations. *J. Econ. Entom.* **78**, 1154-1156 (1985).

10. K. S. Delaplane, J. Van der Steen, E. Guzman-Novoa, Standard methods for estimating strength parameters of Apis mellifera colonies. *J. Apic. Res.* **52**, 1-12 (2013).

11. F. C. Riddell Pearce, M. J. Couvillon, F. L. W. Ratnieks, Hive relocation does not adversely affect honey bee (Hymenoptera: Apidae) foraging. *PSYCHE* **2013**, 693856 (2013).

12. L. Tornqvist, The Bank of Finland's Consumption Price Index. *Bank of Finland Monthly Bulletin* **10**, 1-8 (1936).

13. N. W. Calderone, Evaluating subsampling methods for estimating numbers of varroa jacobsoni mites (Acari: Varroidae) Collected on Sticky-Boards. *J. Econ. Entom.* **92**, 1057-1061 (1999).

14. N. W. Calderone, S. Lin, Rapid determination of the numbers of varroa destructor, a parasitic mite of the honey bee, Apis mellifera, on sticky-board collection devices. *Apidologie* **34**, 11-17 (2003).

15. P. Lesica, P. M. Kittelson, Precipitation and temperature are associated with advanced flowering phenology in a semi-arid grassland. *J. Arid Envir.* **74**, 1013-1017 (2010).

16. M. A. Huston, Precipitation, soils, NPP, and biodiversity: resurrection of Albrecht's curve. *Ecol. Mono.* **82**, 277-296 (2012).

17. P. Q. Craufurd, T. R. Wheeler, Climate change and the flowering time of annual crops. *J. Exper. Bot.* **60**, 2529-2539 (2009).

18. J. L. Stallings, Weather indexes. *J. Farm Econ.* **42**, 180-186 (1960).

19. R. U. Igugo, S. O. Alaku, B. N. Marire, Effects of season on weight gain by honeybee hive (apis Mellifera). *J. Exper. Res.* **4**, 43-47 (2016).

20. L. H. S. Alves, P. C. R. Cassino, F. Prezoto, Effects of abiotic factors on the foraging activity of Apis mellifera Linnaeus, 1758 in inflorescences of Vernonia polyanthes Less (Asteraceae). *Animal Sciences* **37**, 405-409 (2015).

21. K. Tan, S. Yang, Z. W. Wanf, S. E. Radloff, B. P. Oldroyd, Differences in foraging and broodnest temperature in the honey bees Apis cerana and A. mellifera. *Apidologie* **43**, 618-623 (2012).

22. M. D. Cramer, M. T. Hoffman, The consequences of precipitation seasonality for mediterranean-ecosystem vegetation of South Africa. *PLOS ONE* **10**, e0144512 (2015).

23. P. V. R. Reddy, T. Rashmi, A. Varghese, Foraging activity of Indian honey bee, Apis Cerana in relation to ambient climate variables under tropical conditions. *J. Envir. Biol.* **36**, 577-581 (2015).
