# Supplementary material for: Sub-lethal concentrations of neonicotinoid insecticides at the field level affect negatively honey yield: Evidence from a 6-year survey of Greek apiaries
Source: PLoS One. 2019 Apr 25;14(4):e0215363. doi: 10.1371/journal.pone.0215363 (PMC6483167; doi:10.1371/journal.pone.0215363)
Supplement: S1 Table — (PDF) [file pone.0215363.s002.pdf]

Table S1: Summary Statistics of the Variables

Variable	Mean	Min	Max	Std.Dev.
Output and Inputs				
Honey Production (in kgs)	3,160	802	8,508	1,671
Veterinary Expenses (in Euros)	242	74	787	135
Intermediate Inputs (in Euros)	1,867	488	10,761	1,680
Family Labor (in hours)	322	62	1,167	170
Capital Stock (in Euros)	3,076	333	14,507	2,639
Number of Bees (in 000s)	5,322	1,780	14,000	2,525
Bee Farm Characteristics				
Mite Infestation (No of Mites)	5,507	1,872	13,051	1,864
Winter Precipitation (in mm)	422	216	1,072	147
Relative Humidity (%)	0.44	0.24	0.61	0.086
Aridity Index	0.79	0.35	1.49	0.33
Damaging Input				
Neonicotinoids (in $\mu g/kg$)	1.386	0.377	2.842	0.614
