# Supplementary material for: Sub-lethal concentrations of neonicotinoid insecticides at the field level affect negatively honey yield: Evidence from a 6-year survey of Greek apiaries
Source: PLoS One. 2019 Apr 25;14(4):e0215363. doi: 10.1371/journal.pone.0215363 (PMC6483167; doi:10.1371/journal.pone.0215363)
Supplement: S2 Table — (PDF) [file pone.0215363.s003.pdf]

Table S2: Parameter Estimates of the Translog Production Function

Par.	Est.	St. Error	Par.	Est.	St. Error
β_0	0.8572	0.0262**	β_{VV}	0.2970	0.1329**
β_B	0.3059	0.0853**	β_{IL}	-0.1344	0.1053
β_I	0.1661	0.0269**	β_{IC}	-0.0513	0.0693
β_L	0.1701	0.0402**	β_{IV}	0.2521	0.1374*
β_C	0.1080	0.0252**	β_{LC}	0.0112	0.1127
β_V	0.1837	0.0416**	β_{LV}	-0.4706	0.2226**
β_T	0.0698	0.0314**	β_{CV}	-0.3932	0.1208**
β_{TT}	0.1309	0.0606**	β_{BI}	-0.4476	0.1644**
β_{BT}	0.0066	0.0711	β_{BL}	0.9004	0.1975**
β_{IT}	0.0140	0.0321	β_{BC}	0.3735	0.1135**
β_{LT}	-0.0336	0.0424	β_{BV}	0.0977	0.2275
β_{CT}	0.0158	0.0237	α_Z	-0.4988	0.1512**
β_{VT}	0.0143	0.0535	α_{ZM}	-0.0875	0.0504*
β_{BB}	-0.6035	0.2434**	α_{ZP}	0.2468	0.0653**
β_{II}	-0.0261	0.0758	α_{ZH}	-0.0262	0.0817
β_{LL}	0.0372	0.0786	α_{ZA}	0.1402	0.0457**
β_{CC}	0.0426	0.0315	$\bar{R}^2$	0.8848	

B refers to bee density, I to intermediate inputs, L to family labor, C to capital, V to veterinary expenses, T to time, Z to insecticides, M to mite infestation, P to winter precipitation, H to relative humidity, and A to aridity index,. Robust standard errors are reported in the table. * and ** indicate statistical significance at the 10 and 5 per cent level, respectively.
